# Fully endovascular management of late pseudoaneurysm after aortic coarctation repair using graft sealing and coarctation occlusion

**DOI:** 10.1016/j.jvscit.2026.102269

**Published:** 2026-04-21

**Authors:** Estefanía San Ginés Bahíllo, Andrés Álvarez Salgado, Pablo del Canto Peruyera, Manuel Javier Vallina-Victorero

**Affiliations:** Department of Vascular Surgery, Cabueñes University Hospital, Gijón, Spain

**Keywords:** Aortic coarctation, Pseudoaneurysm, Endovascular repair, Extra-anatomic bypass, Amplatzer occluder, Graft complications

## Abstract

We report the case of a 79-year-old woman with aortic coarctation previously repaired by extra-anatomic bypass, who developed a late pseudoaneurysm at the distal graft anastomosis. A fully endovascular approach was used, combining an endograft and an Amplatzer Septal Occluder to seal both the pseudoaneurysm and the patent coarctation. The patient recovered uneventfully and remains stable at 1-year follow-up. This case illustrates the safety and efficacy of endovascular techniques for managing complex late complications after aortic coarctation repair.

Coarctation of the aorta (CoA) is one of the most well-known congenital heart malformations, with limited survival expectancy without surgical treatment.^1^ Most patients undergo intervention during the neonatal period; however, it is not uncommon for surgery to be performed during adolescence or adulthood, often due to poorly controlled hypertension. Currently, a wide variety of surgical techniques are available for treating this condition, including both open surgery and endovascular approaches.^1^, ^2^, ^3^ Traditionally, correction involved different open surgical procedures, which was associated with significant complications even decades after the procedure.^4^^,^^5^ In this context, we present the case of a patient with a history of a partial extra-anatomic bypass performed years earlier, who on recent radiological follow-up showed an image compatible with a pseudoaneurysm at the distal anastomosis of the graft.

## Clinical case

**Fig 1:**
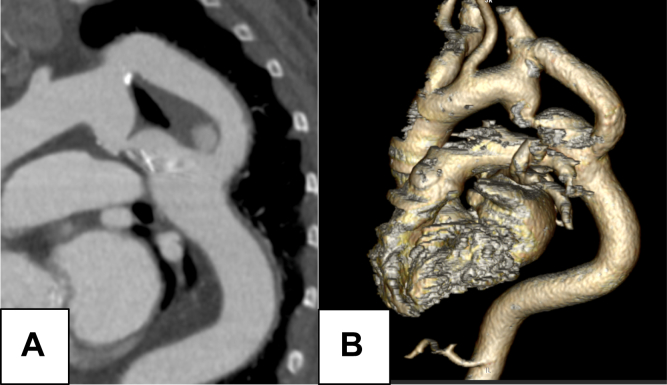
Preoperative CT angiography. Contrast-enhanced CT angiography showing a pseudoaneurysm arising from the distal anastomosis of the extra-anatomic graft. The native coarctation site remains patent and may contribute to retrograde flow into the pseudoaneurysm sac **(A)**. CT angiography reconstruction of aortic coarctation **(B)**. *CT*, Computed tomography.

A 79-year-old woman with a history of CoA repaired at age 42 years due to refractory hypertension. She had undergone a partial extra-anatomic bypass, from the origin of the left subclavian artery to the distal aorta beyond the coarctation, via left lateral thoracotomy. Subsequently, she underwent a second surgery through median sternotomy for a bicuspid aortic valve dysfunction causing left ventricular failure; she received a prosthetic valve and was on anticoagulation therapy, also due to atrial fibrillation. The patient was referred from cardiology clinics after a recent computed tomography angiography scan revealed a suspicious image suggestive of an 11-mm pseudoaneurysm at the distal anastomosis of the graft (Fig 1). Given the high morbidity and mortality associated with this complication, reintervention was decided. Considering the elevated surgical risk due to her previous major surgeries (EuroSCORE 5.97%, American Society of Anesthesiologists IV), an entirely endovascular approach was chosen. Initially, it was thought that sealing the pseudoaneurysm neck with a stent graft at that level would limit flow into the pseudoaneurysm. Nevertheless, the small-caliber residual coarctation (8 × 11 mm) remained patent and continued supplying blood to the pseudoaneurysm. Given this finding, the surgical strategy was designed in two sequential steps: first, complete occlusion of the residual coarctation zone to eliminate the inflow to the pseudoaneurysm; and second, exclusion of the pseudoaneurysm by deploying a customized endograft within the previously implanted graft to seal its neck.

## Surgical technique

**Fig 2:**
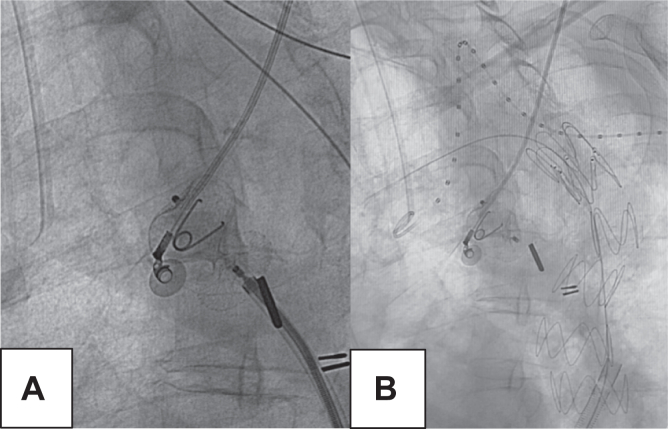
Deployment of the endograft and Amplatzer Septal Occluder placement. Fluoroscopic image demonstrating positioning of a 12-mm Amplatzer Septal Occluder device at the native aortic coarctation site, achieving complete occlusion and preventing retrograde filling of the aneurysm sac **(A)**. Fluoroscopic image demonstrating subsequent deployment of the thoracic endograft, achieving complete sealing of the pseudoaneurysm sac with no evidence of endoleak **(B)**.

Under general anesthesia and via percutaneous access, three access points were obtained: left brachial artery, left common femoral artery, and right common femoral artery. A 4F introducer was placed in the arm, and 8F sheaths were placed in both femoral arteries with the Perclose technique (Abbott Vascular). Local and systemic heparinization was administered. Through the right femoral approach, the ascending aorta was catheterized using an Advantage 0.035″ × 260 mm guidewire, over which a 12-mm Amplatzer Septal Occluder device was advanced and positioned at the coarctation site to seal it (Fig 2, *A*). Subsequently, via the left brachial approach, the descending aorta through the graft was catheterized with an Advantage 0.035″ × 260 mm guidewire, which was snared from the left femoral access. A Berenstein 4F × 65 mm catheter was advanced, and exchange was made for a Back-up Meier stiff guidewire, over which a customized Cook endograft measuring 24 × 30 × 130 mm was deployed. Final remodeling was performed with a compliant balloon (Fig 2, *B*). Control angiography showed no leaks. Percutaneous closure of the femoral access sites was achieved with two Perclose devices on each side, and manual compression was applied at the brachial access site.

## Discussion

Surgical repair of CoA can be associated with significant long-term complications with a high morbidity and mortality rate that can manifest even decades after the initial intervention. Among these, pseudoaneurysm formation is particularly notable, with reported incidences varying widely according to the primary surgical technique.^4^^,^^6^, ^7^, ^8^, ^9^, ^10^, ^11^ The highest rates have been described after patch aortoplasty using synthetic materials, with documented risks of up to 30% in some series.^12^ Endovascular management of pseudoaneurysms represents a minimally invasive alternative with a potentially lower procedural risk. The deployment of endografts has emerged as the treatment of choice in suitable cases, offering effective sealing and reducing the morbidity associated with reoperative surgery.^5^, ^6^, ^7^^,^^11^ Initially, Amplatzer occlusion devices were primarily used for the closure of septal cardiac defects. However, their application has expanded over time, and they are now among the first occlusion systems approved by the Food and Drug Administration for use in peripheral vascular interventions.^4^ In our case, despite the small diameter of the coarctation site, it remained patent, posing a risk of persistent flow into the pseudoaneurysm over time. Consequently, complete sealing was deemed necessary. Considering the size and morphology of the lesion, a commonly used occlusion plug for septal artery fistula closure was selected.

Regarding the endograft selection, the use of a customized medical device in this case was driven by the unique anatomical constraints imposed by the previously implanted extra-anatomic bypass graft. In our patient, the dimensions of the existing conduit required a tailored reverse-tapered 24 × 30 × 130 mm endograft to ensure precise apposition, effective sealing of the pseudoaneurysm neck, and avoidance of device migration or endoleak. Custom-made devices play an increasingly recognized role in the management of complex aortic pathology. In the context of CoA and its long-term complications, where patients often present with prior surgical repairs, unusual vessel morphology, or size mismatches, custom-made devices represent a valuable tool to achieve technically sound endovascular exclusion when conventional devices would be unsuitable.

**Fig 3:**
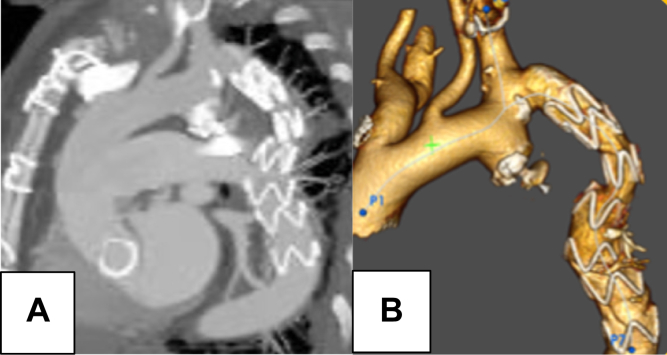
One-year follow-up CT angiography. Follow-up CT scan at 12 months showing stable endograft position, complete thrombosis of the pseudoaneurysm sac, and absence of complications or endoleaks **(A)**. CT angiography reconstruction **(B)**. *CT*, Computed tomography.

Postimplantation imaging confirmed optimal device positioning, complete occlusion of the pseudoaneurysm neck, and effective sealing of the coarctation. The patient was discharged 72 hours after the procedure without any complications and remained stable at 1-year follow-up. Control computed tomography imaging demonstrated correct endograft placement, complete sealing of the pseudoaneurysm, and successful occlusion of the coarctation (Fig 3).

## Conclusions

Surgical correction of CoA is associated with significant long-term risks, necessitating lifelong surveillance. Pseudoaneurysm formation is a serious complication with high morbidity and mortality. Endovascular techniques offer a safer, less invasive alternative with favorable outcomes, especially in patients with elevated surgical risk. These approaches should be considered integral to the management of such complex cases.

## Patient consent

Consent for publication of this case and accompanying images was obtained from the patient.

## Funding

None.

## Disclosures

None.
